# Energy-Adjusted Dietary Inflammatory Index Is Associated With 5-Year All Cause and Cardiovascular Mortality Among Chronic Kidney Disease Patients

**DOI:** 10.3389/fnut.2022.899004

**Published:** 2022-06-14

**Authors:** Ying Huang, Lei Zhang, Mengru Zeng, Fuyou Liu, Lin Sun, Yu Liu, Li Xiao

**Affiliations:** Department of Nephrology, The Second Xiangya Hospital of Central South University, Hunan Key Laboratory of Kidney Disease and Blood Purification, Changsha, China

**Keywords:** energy adjusted dietary inflammation index, 5-year all-cause mortality, 5-year cardiovascular mortality, chronic kidney disease, National Health and Nutrition Examination Survey

## Abstract

**Background:**

Diet management is a pivotal intervention for chronic kidney disease (CKD) patients. Dietary inflammation index (DII) is developed to evaluate the integral inflammatory potential of a diet pattern. However, research about the association between DII and mortality in CKD is limited.

**Objective:**

We conducted a cohort study to investigate the relationship between energy-adjusted DII (E-DII) and the 5-year all-cause and cardiovascular mortality in CKD population.

**Materials and Methods:**

CKD participants with complete E-DII data and death status from National Health and Nutrition Examination Survey (1999–2014) were involved in this study. E-DII was calculated based on dietary recall interviews. Smooth curve fitting, Kaplan–Meier survival analysis, and Cox proportional hazards models were used to evaluate the association between E-DII and the 5-year all cause and cardiovascular mortality. Subgroup analysis was also performed.

**Results:**

A total of 7,207 participants were included (55.46% elderly and 46.54% male) in this study. The 5-year all-cause and cardiovascular mortality were 16.86 and 4.32%, respectively. Smooth curve fitting showed a “J” shape and near linear relationship between the E-DII score and the 5-year all-cause and cardiovascular mortality, respectively. In multivariate Cox proportional hazards models, the hazard ratios (95% confidence intervals [*CI*]) for the highest tertile of the E-DII were 1.33 (1.15, 1.54) for all-cause mortality, and 1.54 (1.15, 2.07) for cardiovascular mortality when compared with the lowest tertile of the E-DII. The subgroup analyses revealed relatively stronger associations between the E-DII and the mortality among CKD patients with other death risk factors.

**Conclusions:**

Energy-adjusted dietary inflammatory index is independently related with the 5-year all-cause and cardiovascular mortality among CKD patients. Therefore, anti-inflammatory diet patterns should be recommended for CKD patients.

## Introduction

Chronic kidney disease (CKD) has become one of the most common health and life-threatening disease. The global prevalence of all-stage CKD and the CKD-associated mortality increased 29.3 and 41.5%, respectively, between 1990 and 2017 (1). High burden of cardiovascular disease and cardiovascular mortality due to volume overload, hypertension, atherosclerosis, and vascular calcification is widely recognized in CKD patients (2).

Inflammation is one of the main systemic nature of CKD, characterized by high levels of proinflammatory cytokines such as tumor necrosis factor (TNF), interleukin 1β (IL-1β), IL-6, and C-reactive protein (CRP) (3, 4). The systematic inflammation is correlated with many adverse events and outcomes in CKD including increase in all-cause and cardiovascular mortality. A prospective cohort study involving 3,875 CKD participants (2–4 stage) investigated the associations between the baseline plasma IL-6, CRP, fibroblast growth factor 23 (FGF23), and the all-cause mortality. Not surprisingly, they found that CKD patients with higher level of IL-6, CRP, and FGF23 suffered 35, 28, and 45% higher risk of death, respectively (5). Our group also demonstrated that platelet-to-lymphocyte ratio, an inflammatory biomarker, is independently associated with an increased 5-year all-cause and cardiovascular mortality in CKD patients (6).

Chronic kidney disease associated inflammation is influenced and regulated by multiple factors including accumulation of uremic toxins and infections like periodontal disease (3). Interestingly, diet is another important source of inflammation. It has been documented that inflammatory cytokines like CRP can be modulated by dietary components and specific nutrients (7). To evaluate the integral anti- and pro-inflammatory effect of diet patterns, dietary inflammatory index (DII) was developed (7). Furthermore, DII score is regarded to be positively associated with numerous inflammatory diseases such as obesity, diabetes, cardiovascular disease, nonalcoholic fatty liver disease, periodontitis, and chronic kidney disease (8–11). However, the correlation between DII and the risk of death among CKD patients has not been elucidated. Therefore, this study aimed to examine the relationship between the E-DII score and the 5-year mortality in CKD population based on the public database National Health and Nutrition Examination Surveys (NHANES). We hypothesized energy-adjusted DII (E-DII) is positively related with the risk of death in CKD population.

## Materials and Methods

### Study Population

The NHANES is a program evaluating the health and nutritional status of the people in the United States. NHANES collects demographics, dietary, medical examination, laboratory, and questionnaire data (12). This program was approved by The National Center for Health Statistics (NCHS) Research Ethics Review Board and the written informed consent was obtained from the participants (13).

**Figure 1:**
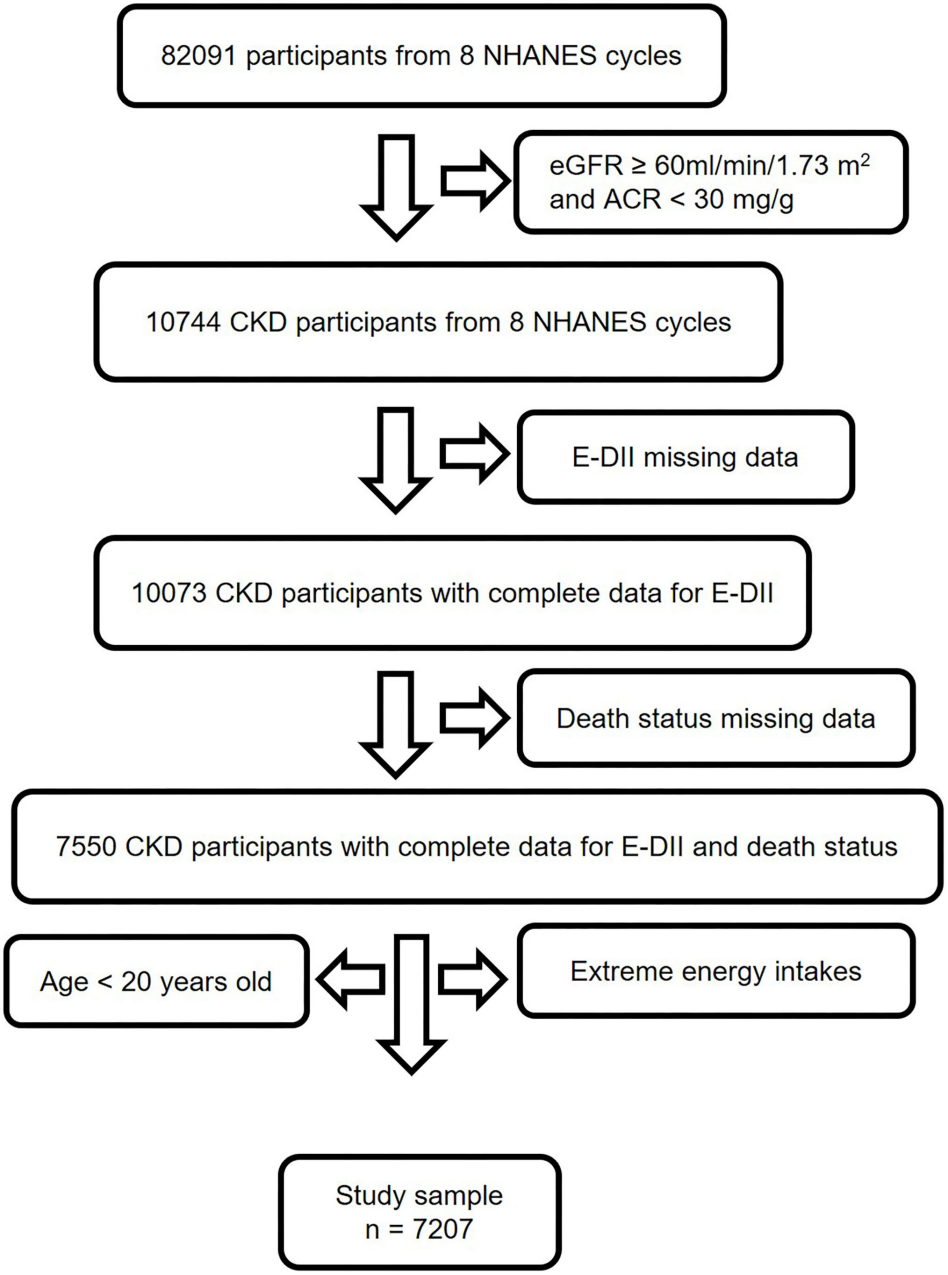
Study flow chart. A total of 82,091 participants from NHANES 1999–2014 were involved in this study. Individuals with eGFR ≥ 60 ml/min/1.73 m^2^, ACR < 30 mg/g and age < 20 years old didn't meet the inclusion criteria. After excluding subjects with missing data for E-DII, mortality, and extreme energy intake, 7,207 participants were finally used for analysis. CKD, chronic kidney disease; NHANES, National Health and Nutrition Examination Survey; E-DII, energy adjusted dietary inflammatory index; eGFR, estimate glomerular filtration rate; ACR, urine albumin to creatinine ratio.

In this study, we collected data from 8 continuous NHANES cycles, ranging from 1999 to 2014 (12). The inclusion criteria were (1) diagnosed with CKD (estimated glomerular filtration rate [eGFR] < 60 ml/min per 1.73 m^2^ or urine albumin-to creatinine ratio (ACR) ≥ 30 mg/g) (14), (2) had complete data for E-DII and death status. While the exclusion criteria were (1) had missing data for E-DII and death status, (2) age < 20 years old, (3) extreme energy intake (< 500 kcal/day or more than 5,000 kcal/day for women, and 8,000 kcal/day for men) (15). As shown in Figure 1, 7,207 participants were involved for analysis.

### Energy-Adjusted DII

Dietary inflammatory index is a method for nutritional evaluation to estimate the influence of diet on systematic inflammatory biomarkers (7). DII is calculated based on 45 different food components' pro- and anti-inflammatory properties. Pro-inflammatory diets display higher and positive DII scores, whereas anti-inflammatory diets show negative DII (7). We collected dietary intake information from 24 h dietary recalls (24 h) in this study. DII scores were calculated based on 27 or 28 food components listed below (16): energy, carbohydrate, protein, fat, dietary fiber, saturated, monounsaturated, and polyunsaturated fatty acids, ω-3 and ω-6 polyunsaturated fatty acids, cholesterol, vitamin A, B1, B2, B3, B6, B12, C, D (missing in NHANES 1999–2006), and E, folic acid, alcohol, beta-carotene, caffeine, iron, magnesium, zinc, and selenium. The details of DII calculation are provided in the Supplementary Material. To minimize the influence of total energy intake, we designed an E-DII (E-DII, DII/total energy intake) as the exposure variable in this study (10).

### Outcome Variable

The outcome of interest was the 5-year all-cause and cardiovascular mortality, which were collected from the NHANES linked mortality file. Reason of death was classified by the NCHS based on the Tenth Revision of International Classification of Diseases (ICD-10). In this study, cardiovascular mortality was defined as death caused by heart and cerebrovascular diseases (6).

### Potential Confounders

Potential confounders included age, gender, race, physical activity, smoking, alcohol consumption, comorbidities, the eGFR categories of chronic kidney disease, and NHANES release cycles. The age was classified to 2 groups: <65 years old (non-elderly) and ≥ 65 years old (elderly). Gender was categorized into “male” and “female.” The race was assigned to 4 groups: Mexican American, non-Hispanic white, non-Hispanic black, and others (17). Physical activity was defined as “inactive” or “active” according to the average physical activity time (“active” was defined as getting more than 75 min/week vigorous or 150 min/week moderate physical activity) (18). Smokers were participants who answered “Yes” for the question “whether smoke more than 100 cigarettes in life.” Alcohol consumers were individuals who drink alcohol more than 0 g/day. NHANES release cycle was categorized as 1999–2000, 2001–2002, 2003–2004, 2005–2006, 2007-2008, 2009-2010, 2011-2012, or 2013-2014. Comorbidities included diabetes, hypertension, overweight, central obesity, dyslipidemia, cardiovascular diseases, and cancer. Diabetes was identified by self-reported physician's diagnosis or application of hypoglycemic drugs or glycated hemoglobin level ≥ 6.5% or fasting blood glucose ≥ 7 mmol/L or blood glucose examined 2 h after Oral Glucose Tolerance Test (OGTT) ≥ 11.1 mmol/L (6). Hypertension was self-reported physician's diagnosis or application of hypertensive drugs or blood pressure ≥ 140/90 mm Hg (6). Overweight was defined as BMI≥ 25 kg/m^2^ (11), central obesity was confirmed by waist circumference ≥ 88 cm (female)/102 cm (male) (19). Dyslipidemia was self-reported physician's diagnosis or application of anti-dyslipidemia drugs or HDL cholesterol level < 1.0 mmol/L (male)/1.3 mmol/L (female) or triacylglycerol ≥ 1.7 mmol/L or LDL cholesterol ≥ 3.0 mmol/L (11, 19). Cancer was recognized by self-reported physician's diagnosis (6). Heart disease was identified by self-reported physician's diagnosis of congestive heart failure or coronary heart disease or angina pectoris or heart attack or stroke (11). CKD eGFR categories: G1, eGFR ≥ 90 ml/min per 1.73 m^2^, G2, eGFR < 90 but ≥ 60 ml/min per 1.73 m^2^, G3, eGFR < 60 but ≥ 30 ml/min per 1.73 m^2^, G4-5, eGFR < 30ml/min per 1.73 m^2^ (20).

### Statistical Analyses

All the analyses were conducted with Empower (R) (21) and RStudio (22). Continuous variable with irregular distribution was described by median and interquartile range (IQR). Categorical variables were presented as frequency and percentage and comparisons among different groups were performed by chi-square tests. Missing values for confounders were set as another level of the categorical covariate. Smooth curve fitting (penalized spline method) was used to evaluate the non-linear association between E-DII and 5-year all cause or cardiovascular mortality. Survival by tertiles of E-DII was determined by the Kaplan–Meier survival analysis, and the log-rank test was used to compare the differences between groups. Then univariate and multivariate Cox proportional hazards models was applied to evaluate the independent association between E-DII and 5-year mortality. In model 1, no covariate was adjusted. In model 2, age, gender, and race were adjusted and in model 3 age, gender, race, physical activity, smoking, alcohol drinking, comorbidities, CKD G categories, and NHANES cycle were adjusted. In model 3, we selected these confounders due to their associations with mortality or a change in effect estimate of more than 10%. We also performed subgroup analyses. Interaction effect was assessed *via* likelihood ration test.

## Results

### Participants Characteristics

The baseline characteristics of participants were summarized in Table 1. In total, 55.46% participants were elderly patients and 46.54% participants were men. The overall E-DII score ranged from −2.51 to 9.00 (Table 1). Participants were equally distributed into 3 groups according to their E-DII score: the first tertile group (T1, E-DII = −2.51 to 0.37), the second one (T2, DII = 0.37 to 1.62), and the third one (T3, DII = 1.62–9.00). Compared with T1, subjects in T3 group tended to be elderly, female, inactive subjects, and non-alcohol consumers. They also had a higher incidence of diabetes, hypertension, central obesity, dyslipidemia, and heart disease than those in T1 group (*p* < 0.05). Notably, participants who consumed more pro-inflammatory diets were more likely to be in CKD G3-5 groups. Besides, blood inflammatory marker CRP concentration increased along with the E-DII (Table 1), suggesting that the dietary inflammatory potential was correlated to systemic inflammation. The overall 5-year all-cause and cardiovascular mortality among CKD patients were 16.86 and 4.32%, respectively. Moreover, the 5-year all-cause and cardiovascular mortality significantly increased in T3 groups, compared with T1.

**Table 1:** Characteristics of 7,207 chronic kidney disease (CKD) patients aged ≥20 years from 8 National Health and Nutrition Examination Survey (NHANES) cycles overall and by tertile of energy adjusted dietary inflammatory index (E–DII).

	**Energy adjusted dietary inflammatory index**				
	**Overall (7207)**	**T1 (2402)**	**T2 (2402)**	**T3 (2403)**	***P*-value**
E-DII (min-max)	−2.51–9.00	−2.51–0.37	0.37–1.62	1.62–9.00	<0.001
Age–over 65 years old, *n* (%)	3997 (55.46%)	1255 (52.25%)	1275 (53.08%)	1467 (61.05%)	<0.001
Gender-male, *n* (%)	3354 (46.54%)	1390 (57.87%)	1157 (48.17%)	807 (33.58%)	<0.001
Race, *n* (%)					<0.001
Mexican American	1067 (14.81%)	375 (15.61%)	322 (13.41%)	370 (15.40%)	
Non-Hispanic white	3818 (52.98%)	1331 (55.41%)	1298 (54.04%)	1189 (49.48%)	
Non-Hispanic black	1484 (20.59%)	417 (17.36%)	503 (20.94%)	564 (23.47%)	
other	838 (11.63%)	279 (11.62%)	279 (11.62%)	280 (11.65%)	
Smoking, *n* (%)	3629 (50.35%)	1198 (49.88%)	1246 (51.87%)	1185 (49.31%)	0.349
Drinking, *n* (%)	1387 (19.25%)	639 (26.60%)	515 (21.44%)	233 (9.70%)	<0.001
Physical activity-active, *n* (%)	3022 (41.93%)	1177 (49.00%)	1007 (41.92%)	838 (34.87%)	<0.001
Diabetes, *n* (%)	2570 (35.66%)	789 (32.85%)	841 (35.01%)	940 (39.12%)	<0.001
Hypertension, *n* (%)	5003 (69.42%)	1572 (65.45%)	1655 (68.90%)	1776 (73.91%)	<0.001
Overweight, (n%)	5149 (71.44%)	1687 (70.23%)	1750 (72.86%)	1712 (71.24%)	0.028
Central obesity, *n* (%)	4530 (66.95%)	1415 (61.82%)	1519 (67.69%)	1596 (71.47%)	<0.001
**Cancer**, ***n*** **(%**)	1147 (15.92%)	421 (17.53%)	369 (15.36%)	357 (14.86%)	0.019
Heart diseases, *n* (%)	1946 (27.00%)	588 (24.48%)	618 (25.73%)	740 (30.79%)	<0.001
Dyslipidaemia, *n* (%)	5187 (72.66%)	1669 (70.01%)	1738 (73.18%)	1780 (74.79%)	<0.001
CKD G category, *n* (%)					<0.001
G1	1655 (22.96%)	625 (26.02%)	549 (22.86%)	481 (20.02%)	
G2	1625 (22.55%)	565 (23.52%)	569 (23.69%)	491 (20.43%)	
G3	3282 (45.54%)	1054 (43.88%)	1067 (44.42%)	1161 (48.31%)	
G4–5	329 (4.57%)	66 (2.75%)	105 (4.37%)	158 (6.58%)	
**NHANES cycles**, ***n*** **(%)**					<0.001
1999–2000	662 (9.19%)	265 (11.03%)	182 (7.58%)	215 (8.95%)	
2001–2002	897 (12.45%)	269 (11.20%)	321 (13.36%)	307 (12.78%)	
2003–2004	888 (12.32%)	265 (11.03%)	337 (14.03%)	286 (11.90%)	
2005–2006	896 (12.43%)	300 (12.49%)	317 (13.20%)	279 (11.61%)	
2007–2008	1089 (15.11%)	332 (13.82%)	332 (13.82%)	425 (17.69%)	
2009–2010	980 (13.60%)	335 (13.95%)	319 (13.28%)	326 (13.57%)	
2011–2012	879 (12.20%)	314 (13.07%)	288 (11.99%)	277 (11.53%)	
2013–2014	916 (12.71%)	322 (13.41%)	306 (12.74%)	288 (11.99%)	
CRP (mg/dL), median (IQR)	0.29 (0.12–0.65)	0.25 (0.10–0.54)	0.30 (0.13–0.66)	0.32 (0.14–0.71)	<0.001
Follow-up time	60 (41–60)	60 (44–60)	60 (40–60)	60 (39–60)	0.079
All-cause death	1215 (16.86%)	330 (13.74%)	408 (16.99%)	477 (19.85%)	<0.001
Cardiovascular death	311 (4.32%)	79 (3.29%)	107 (4.45%)	125 (5.20%)	0.004

*CKD, Chronic Kidney Disease; NHANES, National Health and Nutrition Examination Survey; E–DII, Energy adjusted dietary Inflammatory Index; CRP, Creative reaction protein; T1, Tertile 1; T2, Tertile 2; T3, Tertile 3; IQR, interquartile range; Missing values for total study: physical activity (n = 1631; 22.63%), Smoking (n = 8; 0.11%), hypertension (n = 341; 4.73%), overweight (n = 225; 3.12%), central obesity (n = 441; 6.12%), dyslipidemia (n = 68; 0.94%), cancer (n = 13; 0.18%), heart disease (n = 2; 0.03%), CKD G category (n = 316; 4.38%), CRP (n = 2007; 27.8%)*.

### Energy-Adjusted DII and Mortality

**Figure 2:**
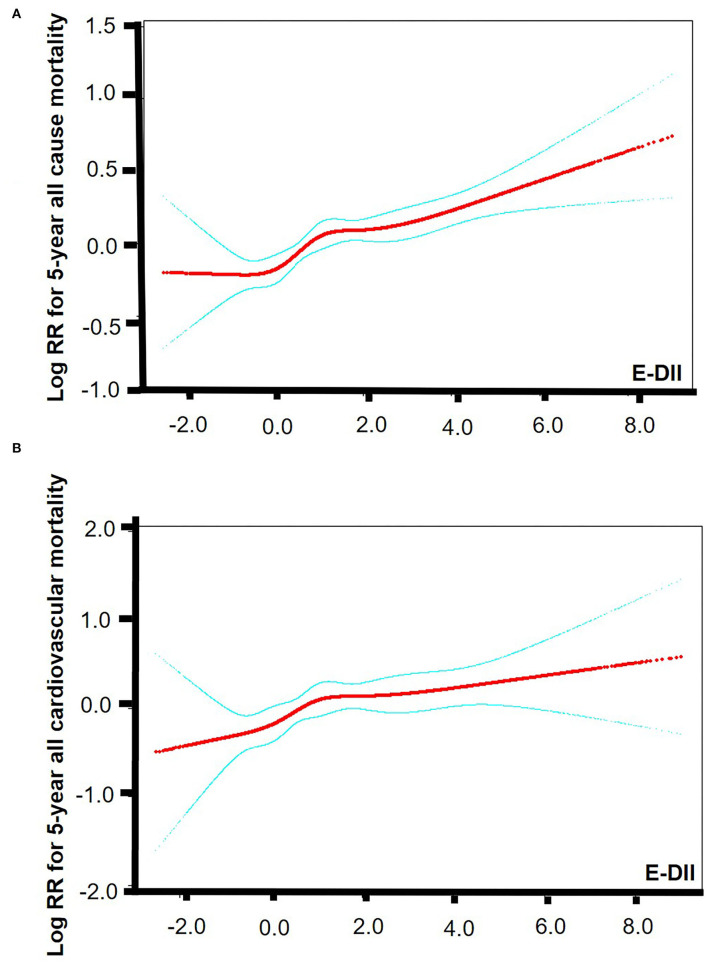
Smooth curve fitting results. **(A)** Smooth curve fitting results between E-DII and 5-year all-cause mortality. **(B)** Smooth curve fitting results between E-DII and 5-year cardiovascular mortality. Risk of death (red) with 95% *CIs* (blue) determined using the Cox proportional Hazard Model. The *p* value for linear is < 0.0001. E-DII, Energy adjusted dietary inflammatory index.

**Figure 3:**
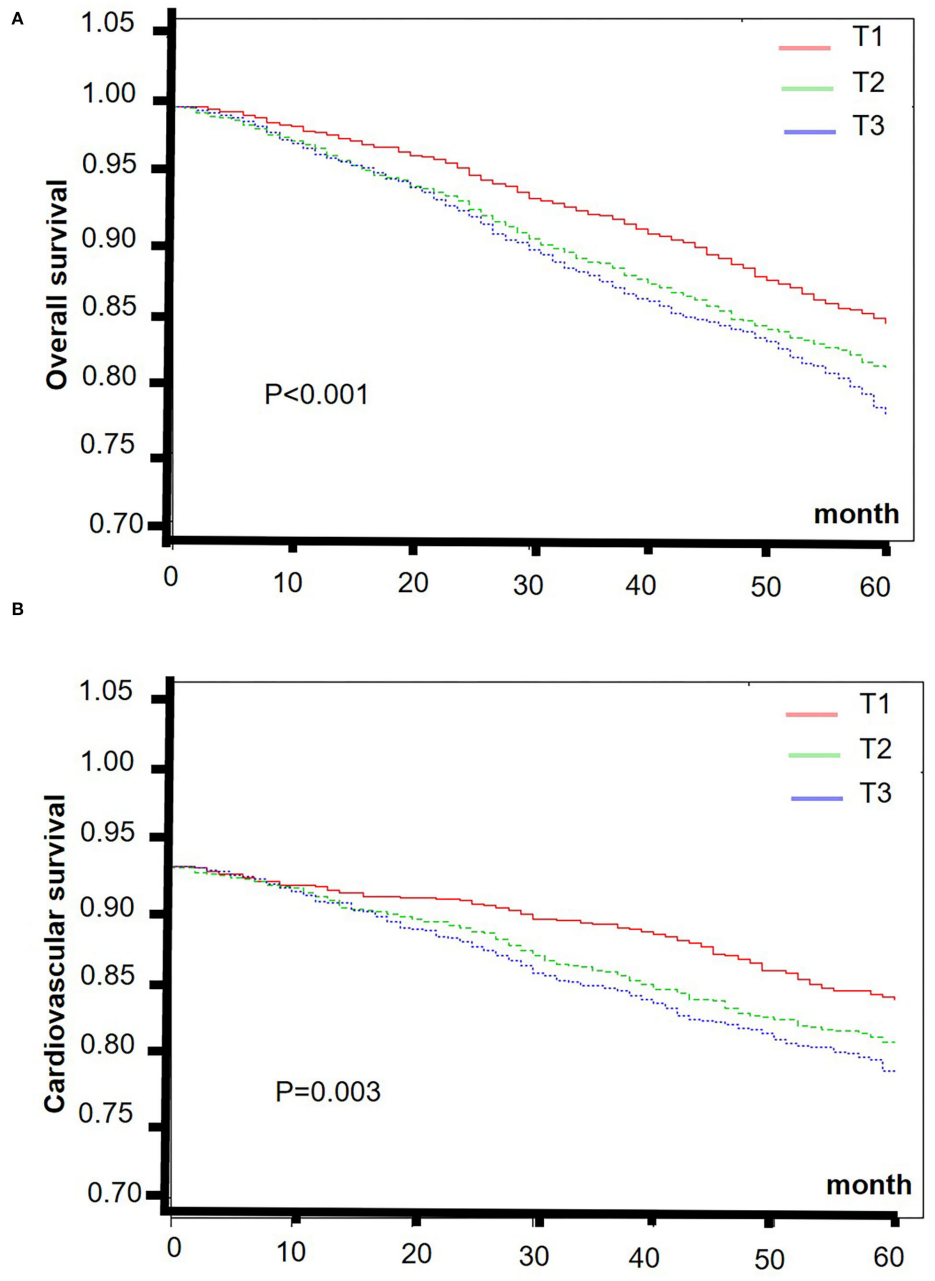
The Kaplan–Meier curves of 5-year overall **(A)** and cardiovascular **(B)** survival rates of the lowest and highest tertiles of E-DII among CKD patients. E-DII, Energy adjusted dietary inflammatory index; CKD, chronic kidney disease.

The smooth curve fitting results showed a “J” shape relationship between E-DII score and the all-cause 5-year mortality and a near-linear association between E-DII and the 5-year cardiovascular mortality without adjustment (Figures 2A,B). The Kaplan–Meier survival curves displayed significant differences in all-cause and cardiovascular survival by tertiles of the E-DII, *p* < 0.05 (Figure 3). Univariate and multivariate Cox proportional hazards model analysis further confirmed that the 5-year all-cause and cardiovascular mortality is positively related with the E-DII scores (Table 2). In crude model, the hazards ratio (*HR*) for 5-year all-cause and cardiovascular mortality were 1.49 (1.29–1.71) and 1.63 (1.23–2.16) for the highest vs. lowest tertile of the E-DII, respectively. Besides, after adjusting for all confounders, the risk of 5-year all-cause mortality increased by 33% in E-DII T3 groups, compared with that in T1 (*p* < 0.001). As expected, the risk of cardiovascular mortality was also enhanced by 54%.

**Table 2:** Associations between the E-DII and mortality among CKD patients aged ≥20 years old.

	**OR (95% CI)**, ***P*****–value**		
	**model 1**	**model 2**	**model 3**
**5–year all–cause mortality**			
T1	1	1	1
T2	1.27 (1.09, 1.46) 0.002	1.32 (1.14, 1.52) <0.001	1.19 (1.03, 1.38) 0.018
T3	1.49 (1.29, 1.71) <0.001	1.53 (1.32,1.76) <0.001	1.33 (1.15, 1.54) <0.001
*p* for trend	<0.001	<0.001	<0.001
**5–year cardiovascular mortality**			
T1	1	1	1
T2	1.39 (1.04, 1.85) 0.028	1.45 (1.09–1.95) 0.012	1.35 (1.00, 1.81) 0.047
T3	1.63 (1.23, 2.16) 0.001	1.70 (1.28–2.27) <0.001	1.54 (1.15, 2.07) 0.004
*p* for trend	<0.001	<0.001	0.004

*Cox proportional hazards models were applied to analyze the association of E–DII and mortality among CKD patients. model 1: no adjustment; model 2: adjusted for age, gender and race; model 3: adjusted for age, gender, race, physical activity, smoking, alcohol drinking, diabetes, hypertension, overweight, central obesity, dyslipidemia, cancer, heart disease, CKD G category and NHANES cycles; CKD, Chronic Kidney Disease; E-DII, Energy adjusted dietary Inflammatory Index; T1, Tertile 1; T2, Tertile 2; T3, Tertile 3; HR, Hazards Ratio; CI, Confidence Interval; NHANES, National Health and Nutrition Examination Survey*.

### Subgroup Analyses

Subgroup analyses results were summarized in Supplementary Tables S2, S3. For the 5-year all-cause mortality, relatively stronger associations between E-DII and mortality were observed among inactive subjects, smokers, patients with dyslipidemia, and patients without cancers. CKD patients in G3-5 also tended to suffer higher risk of death if they adherent to a more pro-inflammatory diet pattern. But no significant interaction effect was identified. Interestingly, the risk of pro-inflammatory diet-associated cardiovascular death was relatively higher in participants who were elderly, female, non-Hispanic White, overweight, with dyslipidemia, with heart disease and without cancers. Notably, compared with CKD G1, patients with worse kidney function were more likely to be influenced by E-DII, *p* for interaction < 0.05.

## Discussion

In this cohort study, we found that E-DII is independently associated with 5-year all cause and cardiovascular mortality. Overall, the results appeared to be more remarkable among CKD patients with other risk factors, including dyslipidemia and worse kidney function.

DII was developed according to the influence of food parameter on blood inflammation markers such as IL-1β/4/6/10, TNF-α, and CRP (7). The previous studies confirmed that DII is positively correlated with TNF-α, IL-1/6/7, and IFN-γ level (23, 24). We and other group also displayed participants with high E-DII scores exhibited increased CRP level (25). Thus, DII is a reliable tool to estimate the dietary inflammatory effect.

The influence of DII on all-cause and cardiovascular death risk has been documented in general population (26, 27), overweight and obese population (28), cancer patients (29, 30), and prediabetics (31). Interestingly, the previous publications reported DII was also associated with renal function, CKD prevalence and progression and CKD-associated complication including hyperparathyroidism (32–34). However, the influence of DII on mortality among CKD patients remained unclear. To our best knowledge, this is the first study to investigate the relationship between DII and the mortality in CKD patients.

The idea of “food as medicine” is widely spread among CKD patients and nephrology physicians (35). Moreover, numerous studies still focus on the diet management in CKD patients. Researchers believe that diet manage could help to delay the progression of kidney function deterioration and prevent bad outcomes. Although limited study about diet management in CKD focused on DII, previous studies could prompt us to connect diet intervention with DII in CKD. Typically, dietary patterns rich in red meat, saturated fats, and simple carbohydrates, higher and positive DII scores (pro-inflammatory diets) (10), while diets composed of vegetables, fruits, whole grains, legumes, nuts, and fish show more negative DII scores (anti-inflammatory diets) (36). The KIDIGO guideline recommended patients with glomerular disease to develop a plant-based diet and restrict sodium, fats and red meat intake (20). An observational cohort study reported that dietary pattern rich in processed and fried foods was independently related with death risk in CKD. In contrast, diets rich in fruits and vegetables tended to be protective (37). A cross-section study involving 2,403 CKD participants also revealed that patients who follow healthy diet patterns would show around 25% lower risk of CKD progression and 24–31% lower all-cause mortality, compared with participants with the lowest adherence (38). Besides, population with medium and high Mediterranean diet (typical anti-inflammatory diet) compliance displayed a 25 and 23% lower mortality risk than population with low compliance (39). Notably, white wine and olive oil, important components of the Mediterranean diet, consumption decreased the plasma levels of CRP and IL-6 in CKD patients (40). All of these reports support our findings on the association between the pro-inflammatory diet adherence and the risk of death.

The mechanisms of the association between DII and the mortality are unknown. Individuals who adhere to diets with high DII score displayed high levels of systematic inflammation biomarkers, such as CRP, IL-6, and TNF-α (7). Inflammation not only increases the death risk directly but also promotes the protein-energy wasting, sarcopenia, vascular calcification and cardiovascular events in CKD patients (41). It was reported that high levels of IL-6, TNF-α, fibrinogen, and albumin increased the risk of the all-cause death and the atherosclerotic vascular events 3.1 fold among CKD patients (4). Besides, proinflammatory diet which is lack of plant fiber, may alter the normal gut microbiome, leading to accumulation of toxic bacterial byproducts. Toxic bacterial byproducts may further promote systemic inflammation, atheromatous changes in arteries, CKD progression and finally adverse outcomes (42, 43).

There are some limitations in this study. Firstly, CKD was judged by one-time UACR and creatinine measurement. Participants might be falsely classified as CKD, especially as early stage of CKD. Secondly, we calculated E-DII based on a recall survey because NHANES didn't perform food frequency questionnaires (FFQs), the most used tool for DII. It may produce recall bias. Besides, participants may underreport their diet information, especially for unhealthy diet components (44). Furthermore, most of the comorbidities were self-reported, which may lead to recall and misclassification bias. Thirdly, participants may change their diet behaviors during follow-up, resulting in over or under-estimation of the association between DII and mortality. Fourthly, DII should be calculated based on 45 food parameters, but only 27 or 28 food components were available in the NHANES data (Vitamin D was missing in NHANES 1999–2006). Although it was demonstrated that DII scores based on 27 or 28 food parameters will not influence its predictive ability (10, 45), we still need to consider the impact on the accuracy. Fifth, we excluded individuals under 20 years and with extreme energy intake. The findings may not be generalized to them. Despite the limitations, there are some strengths in this study. Notably, this is the first time to confirm the relationship between E-DII and mortality among CKD patients based on public NHANES database. The nationwide and non-institutionalized samples make our results more representative. Furthermore, we performed multivariate regression and subgroup analysis to exclude the influence of demographics, behaviors, and other death risk factors. Besides, subgroup analysis revealed that CKD patients with some death risk factors appeared to be easily influenced by inflammatory diets.

## Conclusion

This study demonstrates that E-DII is independently related to the 5-year all-cause and cardiovascular mortality in CKD patients. We should recommend healthy and anti-inflammatory diet patterns to CKD patients to achieve a better outcome.

## Data Availability Statement

Publicly available datasets were analyzed in this study. This data can be found here: https://wwwn.cdc.gov/nchs/nhanes/Default.aspx and https://www.cdc.gov/nchs/data-linkage/mortality-public.htm.

## Ethics Statement

The studies involving human participants were reviewed and approved by the National Center for Health Statistics Research Ethics Review Board. The patients/participants provided their written informed consent to participate in this study.

## Author Contributions

YH, YL, and LX designed the study. YH, LZ, and MZ collected and organized the original data. YH and LZ analyzed the data. YH, MZ, LZ, LS, FL, YL, and LX assisted in the interpretation of the results and writing the manuscript. All authors contributed to the article and approved the submitted manuscript.

## Funding

This work was supported by the National Natural Science Foundation of China [grant numbers 82170744].

## Conflict of Interest

The authors declare that the research was conducted in the absence of any commercial or financial relationships that could be construed as a potential conflict of interest.

## Publisher's Note

All claims expressed in this article are solely those of the authors and do not necessarily represent those of their affiliated organizations, or those of the publisher, the editors and the reviewers. Any product that may be evaluated in this article, or claim that may be made by its manufacturer, is not guaranteed or endorsed by the publisher.
