# Supplementary material for: Energy-Adjusted Dietary Inflammatory Index Is Associated With 5-Year All Cause and Cardiovascular Mortality Among Chronic Kidney Disease Patients
Source: Front Nutr. 2022 Jun 14;9:899004. doi: 10.3389/fnut.2022.899004 (PMC9237483; doi:10.3389/fnut.2022.899004)
Supplement: Supplementary file 1 [file Data_Sheet_1.docx]

DII calculation methods

The calculation of DII followed previous published protocols[1]. Briefly, the individual dietary data was firstly linked to a regionally representative global intake database to compute an inflammatory effect score Z value [individual reported intake- global daily mean intake)/ global standard deviation]. To minimize right skewing, Z value was then converted to a centred percentile score [(2* percentile of Z value -1)]. After that, the centred percentile score of a food parameter is multiplied by the respective inflammatory effect score to obtain a food parameter-specific DII. The individual overall DII score was the sum of 27 or 28 of food parameter-specific DII. All the global daily mean intake and standard deviation, and respective inflammatory effect score were derived from reference[1] and displayed in supplementary table S1.

Table S1. DII calculation methodology and parameter.

| **Food parameter** | **Individual reported intake** | **Global daily mean intake* (units/d)** | **Global daily intake SD*** | **Z value** | **centred percentile score** | **respective inflammatory effect score*** |
| --- | --- | --- | --- | --- | --- | --- |
| Alcohol (g) | A1 | 13·98 | 3·72 | Z1=(A1-13·98)/3.72 | 2* percentile (Z1)-1 | –0·278 |
| Vitamin B12 (μg) | A2 | 5·15 | 2·70 | Z2=(A2-5.15)/2.70 | 2* percentile (Z2)-1 | 0·106 |
| Vitamin B6 (mg) | A3 | 1·47 | 0·74 | Z3=(A3-1.47)/0.74 | 2* percentile (Z3)-1 | –0·365 |
| β-Carotene (μg) | A4 | 3718 | 1720 | Z4=(A4-3718)/1720 | 2* percentile (Z4)-1 | –0·584 |
| Caffeine (g) | A5 | 8·05 | 6·67 | Z5=(A5-8.05)/6.67 | 2* percentile (Z5)-1 | –0·110 |
| Carbohydrate (g) | A6 | 272·2 | 40·0 | Z6=(A6-272.2)/40.0 | 2* percentile (Z6)-1 | 0·097 |
| Cholesterol (mg) | A7 | 279·4 | 51·2 | Z7=(A7-279.4)/51.2 | 2* percentile (Z7)-1 | 0·110 |
| Energy (kcal) | A8 | 2056 | 338 | Z8=(A8-2056)/338 | 2* percentile (Z8)-1 | 0·180 |
| Total fat (g) | A9 | 71·4 | 19·4 | Z9=(A9-71.4)/19.4 | 2* percentile (Z9)-1 | 0·298 |
| Fibre (g) | A10 | 18·8 | 4·9 | Z10=(A10-18.8)/4.9 | 2* percentile (Z10)-1 | –0·663 |
| Folic acid (μg) | A11 | 273·0 | 70·7 | Z11=(A11-273)/70.7 | 2* percentile (Z11)-1 | –0·190 |
| Fe (mg) | A12 | 13·35 | 3·71 | Z12=(A12-13·35)/3.71 | 2* percentile (Z12)-1 | 0·032 |
| Mg (mg) | A13 | 310·1 | 139·4 | Z13=(A13-310.1)/139.4 | 2* percentile (Z13)-1 | –0·484 |
| MUFA (g) | A14 | 27·0 | 6·1 | Z14=(A14-27)/6.1 | 2* percentile (Z14)-1 | –0·009 |
| Niacin (mg) | A15 | 25·90 | 11·77 | Z15=(A15-25.90)/11.77 | 2* percentile (Z15)-1 | –0·246 |
| *n*-3 Fatty acids (g) | A16 | 1·06 | 1·06 | Z16=(A16-1.06)/1.06 | 2* percentile (Z16)-1 | –0·436 |
| *n*-6 Fatty acids (g) | A17 | 10·80 | 7·50 | Z17=(A17-10.80)/7.50 | 2* percentile (Z17)-1 | –0·159 |
| Protein (g) | A18 | 79·4 | 13·9 | Z18=(A18-79.4)/13.9 | 2* percentile (Z18)-1 | 0·021 |
| PUFA (g) | A19 | 13·88 | 3·76 | Z19=(A19-13.88)/3.76 | 2* percentile (Z19)-1 | –0·337 |
| Riboflavin (mg) | A20 | 1·70 | 0·79 | Z20=(A20-1.70)/0.79 | 2* percentile (Z20)-1 | –0·068 |
| Saturated fat (g) | A21 | 28·6 | 8·0 | Z21=(A21-28.6)/8.0 | 2* percentile (Z21)-1 | 0·373 |
| Se (μg) | A22 | 67·0 | 25·1 | Z22=(A22-67.0)/25.1 | 2* percentile (Z22)-1 | –0·191 |
| Thiamin (mg) | A23 | 1·70 | 0·66 | Z23=(A23-1.70)/0.66 | 2* percentile (Z23)-1 | –0·098 |
| Vitamin A (RE) | A24 | 983·9 | 518·6 | Z24=(A24-983.9)/518.6 | 2* percentile (Z24)-1 | –0·401 |
| Vitamin C (mg) | A25 | 118·2 | 43·46 | Z25=(A25-118.2)/43.46 | 2* percentile (Z25)-1 | –0·424 |
| Vitamin D (μg) | A26 | 6·26 | 2·21 | Z26=(A26-6.26)/2.21 | 2* percentile (Z26)-1 | –0·446 |
| Vitamin E (mg) | A27 | 8·73 | 1·49 | Z27=(A27-8.73)/1.49 | 2* percentile (Z27)-1 | –0·419 |
| Zn (mg) | A28 | 9·84 | 2·19 | Z28=(A28-9.84)/2.19 | 2* percentile (Z28)-1 | –0·313 |

RE, retinol equivalents. * Derived from reference [1].

[1] N. Shivappa, S.E. Steck, T.G. Hurley, J.R. Hussey, and J.R. Hébert, Designing and developing a literature-derived, population-based dietary inflammatory index. Public Health Nutr 17 (2014) 1689-96.

Table S2. Subgroup analysis for all-cause mortality

|  |  | **HR** | **95%CI lower** | **95%CI upper** | **P　value** | **p for interaction** |
| --- | --- | --- | --- | --- | --- | --- |
| **Age** |  |  |  |  |  | 0.981 |
| over 65 years old | T2 | 1.20 | 1.02 | 1.41 | 0.030 |  |
|  | T3 | 1.33 | 1.13 | 1.56 | 0.000 |  |
| < 65 years old | T2 | 1.21 | 0.84 | 1.73 | 0.310 |  |
|  | T3 | 1.34 | 0.92 | 1.96 | 0.126 |  |
| **Gender** |  |  |  |  |  | 0.617 |
| male | T2 | 1.27 | 1.06 | 1.52 | 0.011 |  |
|  | T3 | 1.36 | 1.12 | 1.65 | 0.002 |  |
| female | T2 | 1.11 | 0.86 | 1.43 | 0.435 |  |
|  | T3 | 1.28 | 1.01 | 1.62 | 0.038 |  |
| **Race** |  |  |  |  |  | 0.856 |
| Mexican American | T2 | 1.18 | 0.73 | 1.91 | 0.498 |  |
|  | T3 | 1.32 | 0.84 | 2.06 | 0.230 |  |
| Non-Hispanic White | T2 | 1.23 | 1.03 | 1.47 | 0.024 |  |
|  | T3 | 1.32 | 1.10 | 1.59 | 0.003 |  |
| Non-Hispanic Black | T2 | 1.22 | 0.84 | 1.79 | 0.302 |  |
|  | T3 | 1.54 | 1.07 | 2.21 | 0.020 |  |
| other | T2 | 1.04 | 0.57 | 1.89 | 0.900 |  |
|  | T3 | 1.07 | 0.61 | 1.89 | 0.814 |  |
| **Physical activity** |  |  |  |  |  | 0.709 |
| Active | T2 | 1.13 | 0.86 | 1.49 | 0.383 |  |
|  | T3 | 1.33 | 1.00 | 1.77 | 0.054 |  |
| Inactive | T2 | 1.24 | 0.99 | 1.55 | 0.064 |  |
|  | T3 | 1.44 | 1.15 | 1.81 | 0.001 |  |
| **Smoking** |  |  |  |  |  | 1.000 |
| Yes | T2 | 1.21 | 1.00 | 1.46 | 0.046 |  |
|  | T3 | 1.40 | 1.16 | 1.70 | 0.000 |  |
| No | T2 | 1.20 | 0.94 | 1.51 | 0.139 |  |
|  | T3 | 1.24 | 0.99 | 1.57 | 0.065 |  |
| **Drinking** |  |  |  |  |  | 0.856 |
| Yes | T2 | 1.13 | 0.81 | 1.58 | 0.477 |  |
|  | T3 | 1.42 | 0.95 | 2.10 | 0.084 |  |
| No | T2 | 1.20 | 1.02 | 1.42 | 0.030 |  |
|  | T3 | 1.32 | 1.12 | 1.55 | 0.001 |  |
| **Diabetes** |  |  |  |  |  | 0.117 |
| Yes | T2 | 1.35 | 1.07 | 1.70 | 0.011 |  |
|  | T3 | 1.30 | 1.03 | 1.64 | 0.026 |  |
| No | T2 | 1.08 | 0.89 | 1.31 | 0.458 |  |
|  | T3 | 1.36 | 1.12 | 1.65 | 0.002 |  |
| **Hypertension** |  |  |  |  |  | 0.664 |
| Yes | T2 | 1.15 | 0.97 | 1.36 | 0.102 |  |
|  | T3 | 1.32 | 1.12 | 1.55 | 0.001 |  |
| No | T2 | 1.54 | 1.06 | 2.24 | 0.023 |  |
|  | T3 | 1.41 | 0.95 | 2.09 | 0.091 |  |
| **Overweight** |  |  |  |  |  | 0.786 |
| Yes | T2 | 1.18 | 0.98 | 1.43 | 0.085 |  |
|  | T3 | 1.39 | 1.15 | 1.69 | 0.001 |  |
| No | T2 | 1.21 | 0.92 | 1.59 | 0.166 |  |
|  | T3 | 1.35 | 1.04 | 1.77 | 0.027 |  |
| **Central obesity** |  |  |  |  |  | 0.911 |
| Yes | T2 | 1.24 | 1.01 | 1.53 | 0.036 |  |
|  | T3 | 1.35 | 1.10 | 1.65 | 0.004 |  |
| No | T2 | 1.21 | 0.93 | 1.57 | 0.165 |  |
|  | T3 | 1.39 | 1.07 | 1.82 | 0.015 |  |
| **Dyslipidemia** |  |  |  |  |  | 0.269 |
| Yes | T2 | 1.20 | 1.00 | 1.43 | 0.050 |  |
|  | T3 | 1.42 | 1.19 | 1.69 | 0.000 |  |
| No | T2 | 1.28 | 0.97 | 1.68 | 0.077 |  |
|  | T3 | 1.19 | 0.90 | 1.57 | 0.225 |  |
| **Heart disease** |  |  |  |  |  | 0.982 |
| Yes | T2 | 1.17 | 0.94 | 1.45 | 0.151 |  |
|  | T3 | 1.24 | 1.00 | 1.54 | 0.051 |  |
| No | T2 | 1.23 | 1.00 | 1.51 | 0.045 |  |
|  | T3 | 1.34 | 1.09 | 1.65 | 0.005 |  |
| **Cancer** |  |  |  |  |  | 0.136 |
| Yes | T2 | 0.93 | 0.70 | 1.24 | 0.635 |  |
|  | T3 | 1.03 | 0.77 | 1.38 | 0.845 |  |
| No | T2 | 1.32 | 1.11 | 1.57 | 0.002 |  |
|  | T3 | 1.45 | 1.22 | 1.72 | <0.001 |  |
| **CKD G category** |  |  |  |  |  | 0.098 |
| G1 | T2 | 1.06 | 0.68 | 1.65 | 0.800 |  |
|  | T3 | 1.13 | 0.71 | 1.81 | 0.614 |  |
| G2 | T2 | 1.08 | 0.79 | 1.47 | 0.644 |  |
|  | T3 | 1.30 | 0.94 | 1.79 | 0.115 |  |
| G3 | T2 | 1.41 | 1.15 | 1.74 | 0.001 |  |
|  | T3 | 1.39 | 1.13 | 1.71 | 0.002 |  |
| G4-5 | T2 | 0.82 | 0.46 | 1.46 | 0.496 |  |
|  | T3 | 1.47 | 0.87 | 2.48 | 0.150 |  |

The subgroup analysis was adjusted for all presented covariates except effect modifier. Abbreviations: CKD, Chronic Kidney Disease; E-DII, Energy adjusted dietary Inflammatory Index; T1, Tertile 1; T2, Tertile 2; T3, Tertile 3; HR, Hazards Ratio; CI, Confidence Interval; NHANES, National Health and Nutrition Examination Survey.

Table S3. Subgroup analysis for cardiovascular mortality

|  |  | **HR** | **95%CI lower** | **95%CI upper** | **P　value** | **p for interaction** |
| --- | --- | --- | --- | --- | --- | --- |
| **Age** |  |  |  |  |  | 0.356 |
| over 65 years old | T2 | 1.44 | 1.04 | 1.99 | 0.027 |  |
|  | T3 | 1.65 | 1.20 | 2.27 | 0.002 |  |
| < 65 years old | T2 | 0.89 | 0.42 | 1.88 | 0.755 |  |
|  | T3 | 0.86 | 0.37 | 1.96 | 0.712 |  |
| **Gender** |  |  |  |  |  | 0.204 |
| male | T2 | 1.41 | 1.00 | 1.98 | 0.053 |  |
|  | T3 | 1.34 | 0.92 | 1.97 | 0.132 |  |
| female | T2 | 1.31 | 0.73 | 2.33 | 0.362 |  |
|  | T3 | 1.80 | 1.07 | 3.01 | 0.026 |  |
| **Race** |  |  |  |  |  | 0.498 |
| Mexican American | T2 | 0.55 | 0.19 | 1.55 | 0.257 |  |
|  | T3 | 1.45 | 0.61 | 3.43 | 0.399 |  |
| Non-Hispanic White | T2 | 1.61 | 1.11 | 2.33 | 0.011 |  |
|  | T3 | 1.78 | 1.21 | 2.60 | 0.003 |  |
| Non-Hispanic Black | T2 | 1.35 | 0.65 | 2.79 | 0.419 |  |
|  | T3 | 1.61 | 0.80 | 3.26 | 0.181 |  |
| other | T2 | 1.29 | 0.35 | 4.69 | 0.703 |  |
|  | T3 | 0.81 | 0.25 | 2.62 | 0.719 |  |
| **Physical activity** |  |  |  |  |  | 0.567 |
| Active | T2 | 1.28 | 0.72 | 2.27 | 0.403 |  |
|  | T3 | 1.78 | 1.00 | 3.17 | 0.051 |  |
| Inactive | T2 | 1.65 | 1.08 | 2.51 | 0.020 |  |
|  | T3 | 1.74 | 1.14 | 2.67 | 0.011 |  |
| **Smoking** |  |  |  |  |  | 0.810 |
| Yes | T2 | 1.27 | 0.88 | 1.83 | 0.199 |  |
|  | T3 | 1.41 | 0.97 | 2.04 | 0.072 |  |
| No | T2 | 1.54 | 0.93 | 2.57 | 0.096 |  |
|  | T3 | 1.75 | 1.06 | 2.88 | 0.027 |  |
| **Drinking** |  |  |  |  |  | 0.785 |
| Yes | T2 | 1.59 | 0.83 | 3.07 | 0.162 |  |
|  | T3 | 1.94 | 0.91 | 4.16 | 0.087 |  |
| No | T2 | 1.27 | 0.91 | 1.77 | 0.158 |  |
|  | T3 | 1.46 | 1.05 | 2.01 | 0.023 |  |
| **Diabetes** |  |  |  |  |  | 0.499 |
| Yes | T2 | 1.20 | 0.75 | 1.90 | 0.449 |  |
|  | T3 | 1.38 | 0.87 | 2.19 | 0.172 |  |
| No | T2 | 1.42 | 0.96 | 2.09 | 0.078 |  |
|  | T3 | 1.59 | 1.08 | 2.33 | 0.019 |  |
| **Hypertension** |  |  |  |  |  | 0.898 |
| Yes | T2 | 1.40 | 1.01 | 1.95 | 0.044 |  |
|  | T3 | 1.56 | 1.12 | 2.17 | 0.008 |  |
| No | T2 | 1.39 | 0.58 | 3.35 | 0.465 |  |
|  | T3 | 1.47 | 0.64 | 3.38 | 0.359 |  |
| **Overweight** |  |  |  |  |  | 0.958 |
| Yes | T2 | 1.42 | 0.97 | 2.08 | 0.073 |  |
|  | T3 | 1.81 | 1.23 | 2.66 | 0.003 |  |
| No | T2 | 1.12 | 0.65 | 1.94 | 0.683 |  |
|  | T3 | 1.17 | 0.68 | 2.01 | 0.566 |  |
| **Central obesity** |  |  |  |  |  | 0.330 |
| Yes | T2 | 1.60 | 1.06 | 2.40 | 0.024 |  |
|  | T3 | 1.57 | 1.03 | 2.39 | 0.035 |  |
| No | T2 | 1.28 | 0.77 | 2.15 | 0.340 |  |
|  | T3 | 1.52 | 0.90 | 2.57 | 0.117 |  |
| **Dyslipidemia** |  |  |  |  |  | 0.978 |
| Yes | T2 | 1.38 | 0.98 | 1.96 | 0.068 |  |
|  | T3 | 1.66 | 1.17 | 2.36 | 0.004 |  |
| No | T2 | 1.31 | 0.73 | 2.33 | 0.365 |  |
|  | T3 | 1.28 | 0.71 | 2.29 | 0.407 |  |
| **Heart disease** |  |  |  |  |  | 0.921 |
| Yes | T2 | 1.33 | 0.89 | 1.98 | 0.170 |  |
|  | T3 | 1.59 | 1.06 | 2.37 | 0.024 |  |
| No | T2 | 1.36 | 0.88 | 2.11 | 0.167 |  |
|  | T3 | 1.38 | 0.89 | 2.15 | 0.154 |  |
| **Cancer** |  |  |  |  |  | 0.527 |
| Yes | T2 | 0.65 | 0.33 | 1.28 | 0.210 |  |
|  | T3 | 1.06 | 0.55 | 2.04 | 0.855 |  |
| No | T2 | 1.56 | 1.12 | 2.18 | 0.009 |  |
|  | T3 | 1.70 | 1.21 | 2.37 | 0.002 |  |
| **CKD G category** |  |  |  |  |  | 0.025 |
| G1 | T2 | 0.62 | 0.25 | 1.51 | 0.290 |  |
|  | T3 | 0.84 | 0.31 | 2.25 | 0.726 |  |
| G2 | T2 | 1.29 | 0.67 | 2.49 | 0.442 |  |
|  | T3 | 1.87 | 0.96 | 3.65 | 0.067 |  |
| G3 | T2 | 1.77 | 1.19 | 2.63 | 0.005 |  |
|  | T3 | 1.53 | 1.02 | 2.30 | 0.042 |  |
| G4-5 | T2 | 0.88 | 0.19 | 4.10 | 0.872 |  |
|  | T3 | 3.54 | 0.89 | 14.03 | 0.072 |  |

The subgroup analysis was adjusted for all presented covariates except effect modifier. Abbreviations: CKD, Chronic Kidney Disease; E-DII, Energy adjusted dietary Inflammatory Index; T1, Tertile 1; T2, Tertile 2; T3, Tertile 3; HR, Hazards Ratio; CI, Confidence Interval; NHANES, National Health and Nutrition Examination Survey.
